# Caso 1/2020 - Mulher de 55 Anos com Insuficiência Cardíaca após Internação por Diagnóstico Presumido de Infarto do Miocárdio e Insuficiência da Valva Mitral com Rotura de Corda Tendínea

**DOI:** 10.36660/abc.20200024

**Published:** 2020-05-11

**Authors:** Desiderio Favarato, Luiz Alberto Benvenuti

**Affiliations:** HC FM USP São PauloSP Brasil Instituto do Coração (InCor), HC-FMUSP, São Paulo, SP – Brasil

**Keywords:** Insuficiência Cardíaca/fisiopatologia, Prolapso da Valva Mitral/cirurgia, Infarto do Miocárdio, Sepse, Cuidados Pós Operatórios, Choque Cardiogênico, Insuficiência Renal

Paciente do sexo feminino, de 55 anos, procedente de Carapicuíba, SP, era portadora de hipertensão arterial e iniciou dispneia a esforços maiores um ano e meio antes da internação. Em setembro de 2016, apresentou dor precordial de forte intensidade, em aperto, que pouco aliviava com repouso, associada a náuseas. Procurou serviço médico em sua cidade, sendo medicada e liberada para casa. Na manhã seguinte, houve repetição da dor e foi internada. No período de internação, apresentou parada cardíaca, revertida com choque elétrico. A paciente permaneceu internada por 10 dias e recebeu alta com os diagnósticos de infarto agudo do miocárdio e valvopatia mitral.

Após a alta hospitalar, evoluiu com dispneia de classe funcional IV (New York Heart Association), episódios esporádicos de dispneia paroxística noturna e ortopneia, e foi encaminhada para o InCor-HCFMUSP. Estava em uso de AAS, 100 mg/dia; furosemida 40mg, 3 vezes ao dia; captopril 50mg, 3 vezes ao dia; clopidogrel 75mg, 1 vez ao dia; sinvastatina 40mg, 1 vez ao dia.

O exame físico revelou frequência cardíaca de 102 bpm, pressão arterial de 118x86 mmHg; a ausculta pulmonar revelou estertores crepitantes em bases pulmonares; a ausculta cardíaca revelou bulhas rítmicas com sopro sistólico mitral +++/6+; o exame abdominal foi normal, e não havia edema de membro inferior.

O ecocardiograma (28/9/2016) mostrou diâmetro de aorta de 28 mm; do átrio esquerdo, de 47 mm; do ventrículo esquerdo diastólico, de 48 mm, e do ventrículo esquerdo sistólico, de 26 mm; fração de ejeção do ventrículo esquerdo, 77%; espessura de septo, de 11 mm; e, da parede posterior, de 9 mm. A valva mitral apresentava rotura parcial de cordas, eversão para dentro do átrio esquerdo da cúspide posterior, com falha da coaptação entre as cúspides e refluxo excêntrico de grau acentuado. A valva tricúspide também apresentava refluxo acentuado. Estimou-se a pressão sistólica da artéria pulmonar em 62 mmHg.

Os exames laboratoriais (8/11/2016) revelaram os seguintes valores: 4.300.000 hemácias/mm^3^, hemoglobina de 12,3 g/dL, hematócrito 38%, 8110 leucócitos/mm^3^, creatinina de 1,75mg/dL, sódio 140 mEq/L, potássio 3,8 mEq/L.

O eletrocardiograma (8/11/2016) revelou sobrecarga atrial esquerda e atraso final de condução intraventricular do estímulo ( Figura 1 ).

**Figura 1:**
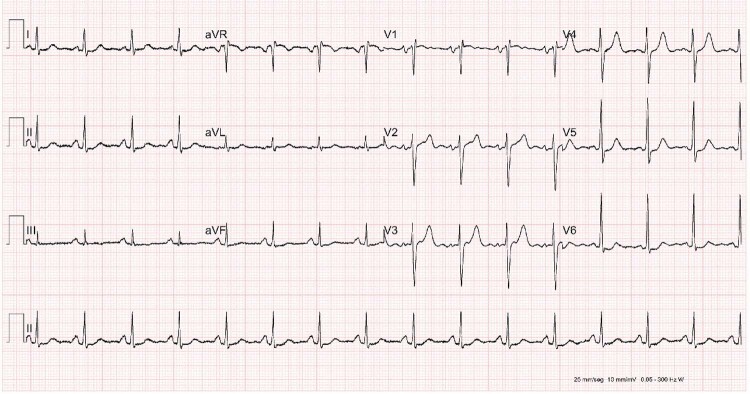
ECG (8/11/2016) evidenciando ritmo sinusal, sobrecarga biatrial e atraso final de condução do estímulo intraventricular.

Havia cardiomegalia acentuada e sinais de congestão pulmonar à radiografia de tórax ( Figura 2 ).

**Figura 2:**
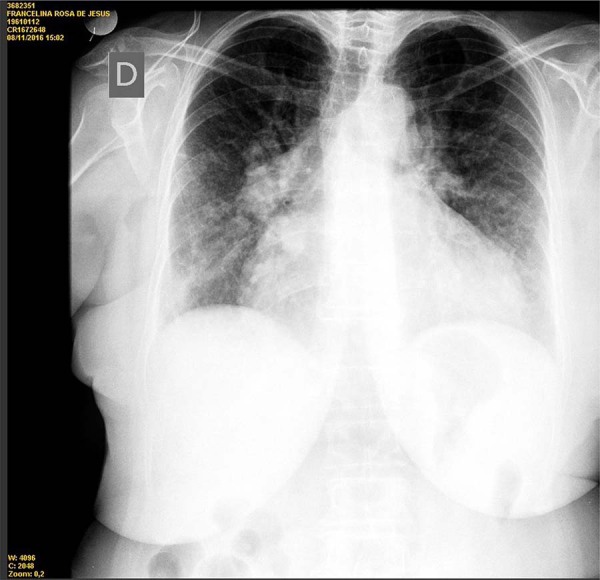
Radiografia de tórax (8/11/2016) mostrando congestão pulmonar e acentuada cardiomegalia.

Foram solicitados ecocardiograma e cateterismo cardíaco com cinecoronariografia.

A cinecoronariografia (8/2/2017) revelou lesão no tronco da coronária esquerda de 40%, duas lesões de 50% no óstio e 90% no terço médio do ramo interventricular anterior; lesão proximal de 60% no ramo circunflexo e lesão de 60% no terço médio da coronária direita ( Figura 3 ).

**Figura 3:**
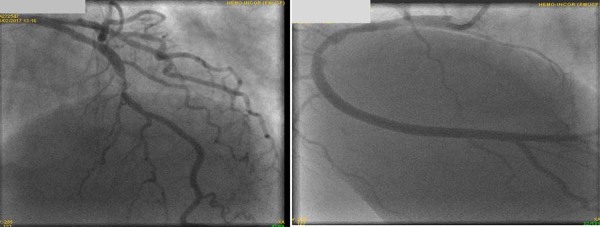
Cinecoronariografia. Painel esquerdo: coronária esquerda em OAD – lesão de interventricular anterior, 80% e 50% proximal de circunflexa. Painel direito: coronária direita – lesão focal de 50%.

Foi indicada cirurgia de correção da insuficiência valvar e de revascularização miocárdica.

Os exames pré-operatórios revelaram: hemácias, 4.300.000/mm^3^; hemoglobina, 12,4 g/dL; hematócrito, 37%; leucócitos, 13.990/mm^3^ (2% bastões, 80% segmentados, 0% eosinófilos, 8% linfócitos e 10% monócitos); plaquetas, 173.000/mm^3^; colesterol total, 158 mg/dL; HDL-C, 28 mg/dL; LDL-C, 109 mg/dL; triglicérides, 103 mg/dL; creatina fosfoquinase (CPK), 2938 U/L; glicose, 120 mg/dL; ureia, 310 mg/dL; creatinina, 4,79 mg/dL; sódio, 136 mEq; potássio, 4,9 mEq/L; alanina aminotransferase (AST), 988 U/L; aspartato aminotransferase (ALT), 681 U/L; ácido úrico, 27,1 mg/dL; hemoglobina glicada, 5,4%; urina I com proteinúria de 0,38 g/L e sedimento com 14.000 células epiteliais/mL, 63.000 leucócitos/mL e 4290 cilindros hialinos/mL; TSH, 6,25 *µ* UI/mL; T4 livre, 0,98 mg/dL; tempo de trombina (INR), 1,4; relação de tempos de tromboplastina parcial ativada (TTPA) de 0,96. Os resultados da sorologia para hepatites B e C e para HIV foram negativos.

Em vista dessas alterações laboratoriais, a paciente foi convocada à Emergência do InCor (23/2/2017).

A paciente referiu que, após realização de cateterismo cardíaco em 8/2/2017, recebeu prescrição de atorvastatina, e desde então vinha evoluindo com mialgia difusa e piora de classe funcional, com dispneia ao repouso e ortopneia até 3 dias antes da internação, associadas a redução do débito urinário, com urina de coloração escurecida. Tinha também dor torácica na região inframamária, com irradiação para a região epigástrica e piora aos esforços habituais, mal caracterizada com duração de horas e sem fatores de melhora. Negou febre e tosse. Referia estar obstipada havia 3 dias.

Ao exame físico, a paciente apresentava-se em estado geral regular, descorada 2+/4+, hidratada, ictérica +/4+, acianótica e afebril. A frequência cardíaca era de 65 bpm; a pressão arterial, de 70x50 mmHg; a saturação de oxigênio, de 90%, e havia crepitações finas em bases pulmonares; a ausculta cardíaca revelou bulhas rítmicas, e sopro holossistólico regurgitativo 3+/6+ em foco mitral; o abdome estava plano, com presença de ruídos hidroaéreos e com fígado palpável a 2 cm do rebordo costal direito; não havia edema nem sinais de trombose venosa profunda em membro inferior.

Diagnosticaram-se choque cardiogênico e rabdomiólise, insuficiência renal aguda, hepatite isquêmica e possível endocardite infecciosa. Foram prescritos noradrenalina; furosemida 40 mg intravenosa de 8 em 8h; e antibióticos ceftriaxona e oxacilina.

Os exames laboratoriais (23/2/2017) revelaram: hemoglobina, 12 g/dL; hematócrito, 35%; leucócitos, 14.230/mm^3^ (82% neutrófilos, 8% linfócitos e 10% monócitos); 147.000 plaquetas/m^3^; CK-MB massa, 54,5ng/dL; troponina I, 0,349 ng/mL; ureia, 313 mg/dL; creatinina, 4,94 mg/dL; AST, 938 U/L; ALT, 746 U/L; gama-glutamil transferase (gama GT,) 473 U/L; fosfatase alcalina (FA), 279 U/L; proteína total do soro, 7 g/dL; bilirrubinas totais, 1,67 mg/dL; bilirrubina direta, 1,15 gm/dL; lipase, 799 U/L; proteína C reativa (PCR), 98,69 mg/L. O tempo de protrombina (INR) foi de 1,4 e a relação dos tempos de trombina parcial ativada (TTPA) foi de 0,96. A urina revelou hemoglobina livre ++, leucócitos 32.000/mL, eritrócitos 13.000/mL, sem cilindrúria. A gasometria revelou pH de 7,40; pCO_2_ de 18,7 mmHg; pO_2_ de 99,9 mmHg; saturação de O_2_ de 99,9%; e bicarbonato de 11,2 mmol/L. O valor do lactato foi de 49 mg/dL.

A cultura de sangue foi positiva para *Staphylococcus hominis* sensível a oxacilina, e a urocultura foi positiva para *Eschericchia coli* multissensível. Foram então trocados, no dia 3/3/2017, os antibióticos para vancomicina, piperacilina e tazobactam. Além da administração de fármacos vasoativos, foi realizada hemodiálise.

O ecocardiograma transtorácico não revelou vegetações, e estimou-se a fração de ejeção do ventrículo esquerdo em 65%, sem alteração da motilidade segmentar. A valva mitral apresentava prolapso da cúspide posterior, com sinais de ruptura de corda associada. O estudo com Doppler e mapeamento com fluxo em cores demonstraram insuficiência de grau acentuado (jato excêntrico).

Os exames laboratoriais (2/3/2017) revelaram: hemoglobina, 9,2 g/dL; hematócrito, 28%; leucócitos, 12.220/mm^3^ (1% bastões, 88% segmentados, 5% linfócitos e 6% monócitos); plaquetas, 123.000/mm^3^; ureia, 54 mg/dL; creatinina, 1,68 mg/dL; sódio, 139 mEq/L; potássio, 3,0 mEq/L; AST, 44 U/L; ALT, 160 U/L.

Foi submetida (3/3/2017) a cirurgia com plástica de valva mitral com reconstrução e comissurotomia sem anuloplastia (ressecção quandrangular), fechamento de comunicação interatrial e revascularização miocárdica com enxerto da artéria torácica interna esquerda (mamária esquerda) para interventricular anterior e ponte de safena para descendente posterior da direita, além de fechamento de comunicação interatrial.

O ecocardiograma no pós-operatório imediato (3/3/2017) revelou discreta insuficiência mitral.

A radiografia de tórax (3/3/2017) no leito em pós-operatório imediato revelou eletrodos de monitoração cardíaca, cateter venoso central, dreno pleural em hemitórax esquerdo, sutura metálica esternal, campos pulmonares livres e área cardíaca normal.

A biopsia do folheto posterior da valva mitral revelou fibrose e acentuada degeneração mucoide do estroma da cúspide (B17-0412).

A paciente apresentou convulsão no dia 4/3/2017 e iniciou tratamento com lamotrigina. A tomografia de crânio não revelou alterações.

O ecocardiograma (20/3/2017) revelou: valva mitral com redução da coaptação de suas cúspides, cúspide posterior com discreta calcificação e redução da sua mobilidade, e discreta insuficiência. Não foram observadas imagens de trombos em átrios e respectivos apêndices ou imagens sugestivas de vegetações.

Recebeu alta em 29/3/2017, e três dias depois veio à Emergência com queixas de dificuldade para alimentar-se, com episódios de vômitos, apesar do uso de ondansetrona, e diarreia. Queixou-se também de rouquidão e zumbido nas duas orelhas. Negou tontura ou vertigem. Negou febre. Referia dispneia ao repouso desde 1 dia antes.

Estava em uso de amiodarona 200mg, 1 vez ao dia; AAS 100mg, dia, lamotrigina, 25mg/dia; furosemida 40mg, 1 vez ao dia; ondansetrona 8mg, 3 vezes ao dia; omeprazol 20mg, 1 vez ao dia; dipirona 500mg, 4 vezes ao dia. (Ao exame físico, mostrava-se descorada 3+/4+. A pressão arterial era 94x68 mmHg e a frequência cardíaca era de 64 bpm; a ausculta pulmonar foi normal, e a cardíaca revelou sopro sistólico mitral 2+/6+, abdome e membros inferiores sem alterações.

A radiografia (1^o^/4/2017) revelou focos de condensação para-hilar e borda cardíaca direita, velamento de seios costofrênicos e cardiomegalia global +++, com desdobramento do arco médio esquerdo e luxação do brônquio-fonte esquerdo ( Figura 4 ).

**Figura 4:**
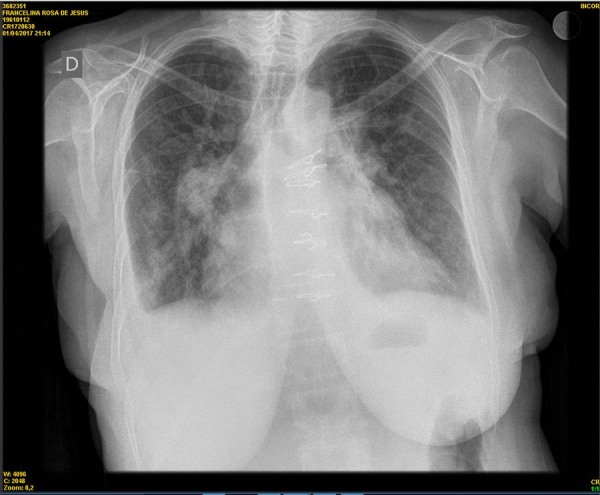
Radiografia de tórax – condensações para-hilares direitas e borda cardíaca direita, sinais de congestão pulmonar e cardiomegalia.

O ECG (2/4/2017) revelou: ritmo sinusal, sobrecarga atrial esquerda, bloqueio de ramo direito e alterações de repolarização ventricular ( Figura 5 ).

**Figura 5:**
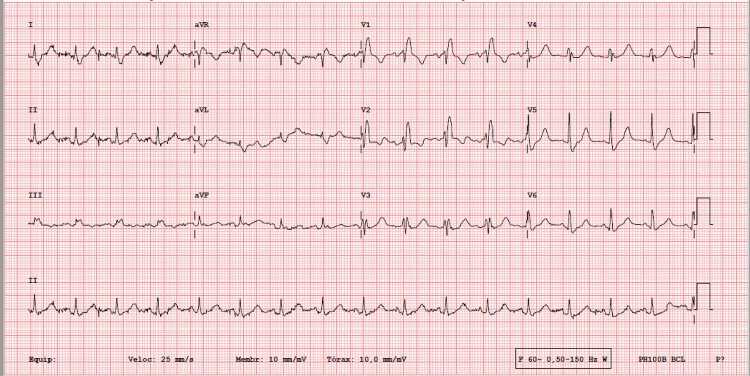
ECG mostrando sobrecarga atrial esquerda, bloqueio de ramo direito.

Os exames laboratoriais revelaram os seguintes valores: hemoglobina, 12,3 g/dL; hematócrito, 37%; leucócitos, 4980/mm^3^; plaquetas, 213.000/mm^3^; proteína C reativa, 23,87mg/L; creatinina, 1,56 mg/dL; ureia, 107 mg/dL; sódio, 134 meq/L; e potássio, 3,4 mEq/L.

O ecocardiograma (4/4/2017) revelou ventrículo esquerdo de dimensões e função sistólicas preservadas, sem alterações segmentares. A valva mitral apresentava acentuada insuficiência, com redução da mobilidade da cúspide posterior, mas sem causar estenose. Foi observada estrutura filamentar na base da cúspide posterior. A valva tricúspide apresentava insuficiência acentuada, em alterações das cúspides valvares. Estimou-se a pressão arterial sistólica pulmonar em 75 mmHg.

A tomografia de tórax revelou foco de condensação em segmento anterior do lobo superior direito e áreas de atelectasias e derrame pleural em ambas as bases pulmonares.

O ecocardiograma transesofágico (7/4/2017) foi semelhante ao transtorácico do dia 4/4/2017.

A paciente recebeu antibioticoterapia com vancomicina e meropeném; houve melhora clínica e recebeu alta em 19 de abril de 2017.

No dia 21/5/2017, voltou à Emergência do InCor por piora da dispneia, agora com ortopneia e edema dos membros inferiores. Além disso, apresentava anúria desde 1 dia antes. Queixava-se também de vômitos diários e diarreia desde a alta da segunda internação. Cinco dias antes dessa internação, havia sido atendida em consulta ambulatorial e, por suspeita de colite pseudomembranosa, recebeu prescrição de ciprofloxacino e metronidazol.

O exame físico revelou frequência respiratória de 22 incursões por minuto, frequência cardíaca de 134 bpm, pressão arterial de 105x80 mmHg, saturação de oxigênio de 98% em uso de 2L/min de O_2_. Ausculta pulmonar mostrava estertores crepitantes até o terço médio; o ritmo cardíaco era regular em dois tempos, sem sopros; o abdome apresentava discreta distensão, com presença de ruídos hidroaéreos; havia edema dos membros inferiores, bilateral +++/4+, sem sinais de trombose venosa profunda.

A radiografia de tórax (21/5/2017) revelou campos pulmonares livres e cardiomegalia ( Figura 6 ).

**Figura 6:**
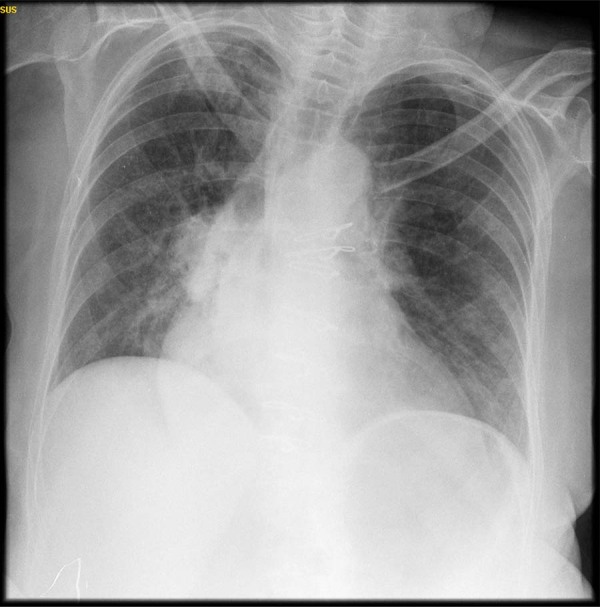
Radiografia de tórax (21/5/2017) mostrando campos pulmonares livres e cardiomegalia.

Os exames laboratoriais revelaram: hemoglobina, 8,2 g/dL; hematócrito, 25%; leucócitos, 22.750/mm^3^ (94% neutrófilos, 3% linfócitos, 3% monócitos); plaquetas, 217.000/mm^3^; ureia, 147 mg/dL; creatinina, 3,21 mg/dL; proteína C reativa, 49,19 mg/L; sódio, 133 mEq/L; potássio, 3,7 mEq/L; lactato, 34 mg/dL.

Apresentou episódio convulsivo e recebeu fenitoína.

Houve assistolia, a qual não respondeu às manobras de reanimação, e a paciente veio a óbito (22h55 min de 21/5/2017).

## Aspectos clínicos

Paciente de 55 anos, com hipertensão arterial e insuficiência cardíaca há um ano e com dor precordial e parada cardiorrespiratória havia cinco meses, evoluiu com insuficiência cardíaca e insuficiência mitral por prolapso de valva mitral e rotura de cordas.

A impressão diagnóstica é de que a insuficiência cardíaca por valvopatia mitral precedeu o episódio de dor precordial e dispneia intensa.

Quanto ao episódio de dor precordial, discute-se se teria sido realmente infarto do miocárdio ou episódio de rotura de corda tendínea com agravamento súbito da insuficiência mitral e edema agudo de pulmão.

A mais recente classificação de infarto do miocárdio (a quarta definição universal) introduziu um novo conceito: *myocardial injury* – lesão miocárdica sem infarto, na qual ocorre elevação de marcador de lesão (a troponina), mas sem configurar infarto, pela inexistência concomitante de quadro clínico sugestivo de alterações eletrocardiográficas e de motilidade de parede. Tal situação pode estar presente em grande número de estados clínicos – anemia, taquicardia ventricular, insuficiência cardíaca, doença renal, hipotensão e choque, hipoxemia. A nova definição incluiu dois tipos de lesão miocárdica: a aguda, com curva de troponinas, elevação e queda; e a crônica, com elevação mantida da troponina.^1^

Na mesma publicação, nota-se que há um contínuo entre “lesão miocárdica isolada” e infarto tipo 2, pois as condições que os originam são as mesmas, dependendo apenas da intensidade e da ocorrência das alterações eletrocardiográficas e ecocardiográficas e de quadro clínico compatível com diagnóstico de infarto.

Nesse sentido, há relato de caso de paciente que procurou Serviço de Emergência por desconforto abdominal e dispneia intensa. O quadro foi precedido de dor precordial 24 horas antes. O paciente já tinha diagnóstico prévio de prolapso de valva mitral. Houve elevação de troponina e alterações inespecíficas da repolarização ventricular. O ecocardiograma revelou insuficiência mitral acentuada e rotura de corda tendínea e prolapso acentuado de metade da cúspide posterior. Diferentemente do caso atual, não havia alterações na coronariografia. Foram feitos os diagnósticos de edema agudo dos pulmões e insuficiência mitral acentuada, provavelmente aguda, por rotura de cordas tendíneas.^2^

Assim, no caso atual, apesar da presença de lesões críticas em coronárias, o evento descrito como infarto agudo do miocárdio pode ter sido somente elevação de marcadores de lesão cardíaca na ausência de infarto.

Embora o prolapso de valva mitral geralmente seja associado a baixo risco de complicações cardiovasculares, algumas publicações põem em dúvida esta crença. Avierinos et al.,^3^ em estudo populacional do Condado de Olmsted, Minnesota, encontraram como fatores de risco primários para mortalidade cardiovascular: insuficiência mitral moderada ou acentuada e disfunção ventricular esquerda, a primeira maior que a segunda. Insuficiência mitral leve, aumento atrial esquerdo, uma cúspide prolapsada, fibrilação atrial e idade acima de 50 anos foram considerados fatores secundários de risco. Nesse estudo, a morbidade cardiovascular foi de 30%, a mortalidade geral foi de 19% e a cardiovascular 9% em 10 anos de seguimento.^3^ No estudo Framingham, 25% dos portadores de prolapso de valva mitral desenvolveram insuficiência mitral significativa ou necessitaram de cirurgia em um período de 3 a 16 anos.^4^

Rotura de corda tendínea valvar é o fator mais comum de insuficiência mitral aguda, e suas causas mais frequentes são endocardite infecciosa, degeneração mixomatosa e prolapso de valva mitral, que, todavia, podem ocorrer na presença de valvopatia reumática, traumatismo torácico e doença aterosclerótica do coração.^3 , 5 , 6^

No caso atual, as alterações tissulares do próprio prolapso podem ser as causadoras da rotura; entretanto, deve-se sempre afastar endocardite infecciosa nesse tipo de complicação.

O diagnóstico de endocardite infecciosa baseia-se em aspectos clínicos, laboratoriais e de imagem por ecocardiograma. Os critérios de Duke são os recomendados. O diagnóstico é feito na presença de 2 critérios principais ou 1 principal e 3 secundários, ou ainda 5 critérios secundários. São considerados critérios principais: hemocultura positiva para endocardite (duas culturas em intervalo de 12h ou 3 culturas em duas coletas de 1h de diferença para micro-organismos comumente relacionados a endocardite – *Streptoccus viridans, Streptoccus bovis* , *Staphylococcus aureus* , ou grupo HACEK, ou uma cultura de *Coxiella burnetii* ).

Além da hemocultura, são considerados critérios principais as evidências de acometimento endocárdico ao ecocardiograma (preferencialmente transesofágico): massa intracardíaca oscilante em valva ou suas estruturas de suporte; abscesso em anel valvar, regurgitação nova ou intensificada.

Estão entre os critérios secundários: predisposição – valvopatia prévia uso de drogas injetáveis ou de cateteres venosos; elevação de marcadores de inflamação; esplenomegalia; hematúria; púrpura; febre acima de 38ºC; fenômenos vasculares (embolia arterial, infarto séptico pulmonar, aneurisma micótico, hemorragia intracraniana ou conjuntival e lesões de Janeway); fenômenos imunológicos (glomerulonefrite, nódulos de Osler, manchas de Roth e elevação do fator reumatoide); hemocultura positiva por micro-organismos geralmente não associados a endocardite.^7^

No caso atual não foram detectadas vegetações ao ecocardiograma, não havia febre e na hemocultura cresceu cepa de estafilococo não habitualmente associado a endocardite. Além disso, na análise histopatológica dos fragmentos valvares retirados durante a cirurgia não havia evidência de endocardite. Assim, o diagnóstico de endocardite infecciosa pode ser afastado.

Esse paciente apresentou rabdomiólise com o uso de estatina ou por isquemia grave, pois, além da elevação da creatina quinase (CK), também havia elevação de enzimas hepáticas sugestivas de hepatite isquêmica.

A apresentação da rabdomiólise foi clássica com as presenças de dores musculares, fraqueza, urina escura e acentuada elevação da creatina quinase (CK). Também sua complicação mais comum, a insuficiência renal aguda, esteve presente.

A sinvastatina e a atorvastatina são metabolizadas pela CYP3A4 (a isoenzima mais comum da citocromo P_450_), enquanto a rosuvastatina é metabolizada pela CYP2A9. Assim, as primeiras são mais susceptíveis a interações medicamentosas que aumentam as concentrações no plasma e a probabilidade de toxicidade. Sintomas musculares são queixas que variam de 1% a 10% dos pacientes em uso de estatinas, mas em menos de 1% há elevação da CK.^8^

A hepatite isquêmica é caracterizada por insuficiência cardiopulmonar ou circulatória, associada ou não a hipotensão arterial, elevação maciça e reversível das enzimas hepáticas aminotransferases (AST e ALT) e exclusão de outras causas de lesão hepática grave, tais como intoxicação por acetaminofeno, hepatite viral ou outra hepatite tóxica. No caso atual, não se pode afastar lesão hepática por estatina, não houve aumento dos tempos de protrombina com iNR acima de 1,5 e a relação de tempos no TTPA foi normal, alterações presentes na hepatite isquêmica.^9^

A fase final da doença dessa paciente foi decorrente de septicemia, que poderia ser decorrente da infecção por *Clostridium difficile* produtor de toxinas. Só as cepas produtoras da toxina B (TcdB) causam a infecção, mas algumas cepas produzem também a toxina A (TcdA). Elas atuam por inativação da via das Rho GTPases por glicosilação do resíduo de treonina, o que leva à despolimerização da actina e a morte celular e estimula a cascata de inflamação responsável por maior dano tissular, diarreia e colite pseudomembranosa.

O uso de antibióticos pode levar ao desequilíbrio do microbioma intestinal, com diminuição dos Bacteroides e Firmicutes, permitindo a proliferação do *Clostridium difficile.* Lembremos que a paciente recebeu antibióticos de amplo espectro por tempo prolongado.^10^ **(Dr.** **Desiderio Favarato)**

**Hipótese diagnóstica:** insuficiência mitral por rotura de corda tendínea em prolapso de valva mitral, septicemia e falência de múltiplos órgãos. **(Dr.** **Desiderio Favarato)** .

## Necropsia

**Figura 7:**
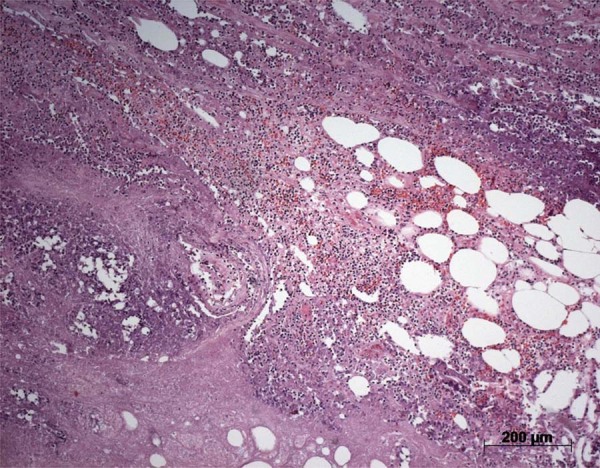
Corte histológico do tecido subcutâneo da região de sutura da safenectomia exibindo intenso processo inflamatório agudo e purulento com áreas de necrose tecidual. Coloração por hematoxilina e eosina.

**Figura 8:**
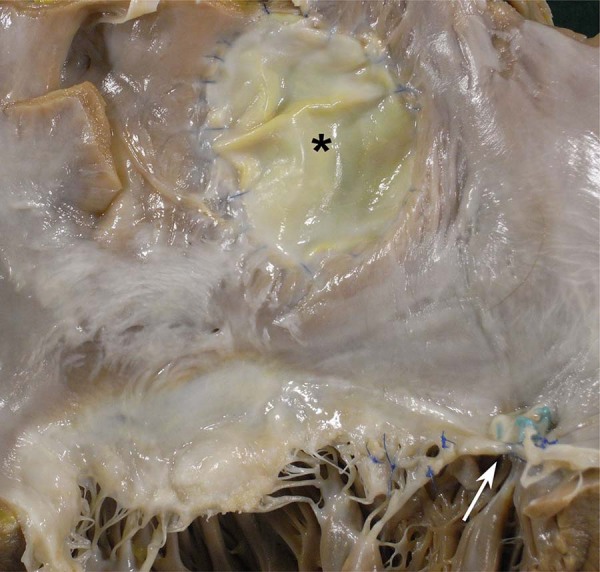
Vista do átrio esquerdo aberto e valva mitral. Note o patch fechando adequadamente a grande comunicação interatrial (asterisco), e sutura cirúrgica

**Figura 9:**
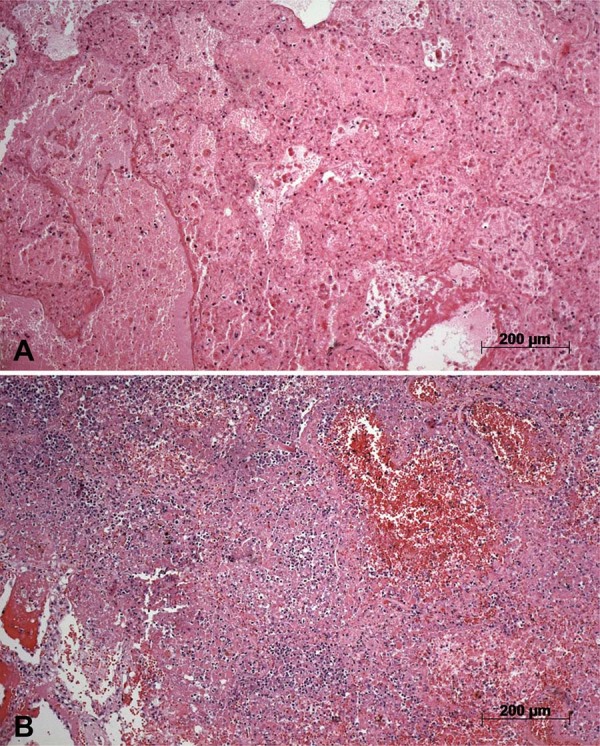
Cortes histológicos dos pulmões exibindo área de infarto pulmonar (A), com apagamento da estrutura tecidual, extravasamento de fibrina e hemorragia, e área de infarto séptico (B), com extenso infiltrado inflamatório purulento semelhante ao exposto na Figura 7. Coloração por hematoxilina e eosina.

**Figura 10:**
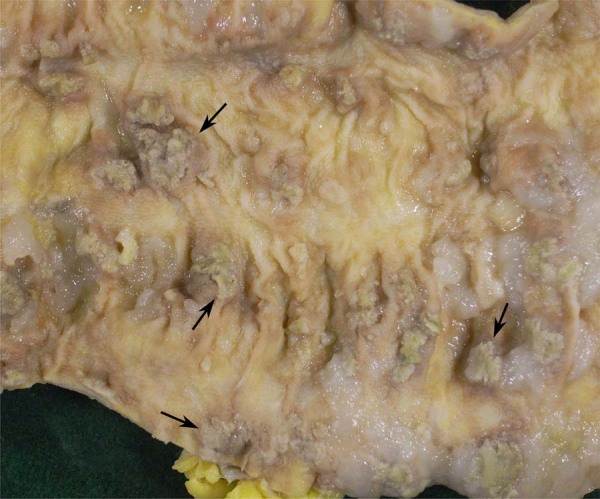
Visão macroscópica do cólon com múltiplas pseudomembranas acastanhadas e granulosas (setas) recobrindo a mucosa de forma multifocal.

**Figura 11:**
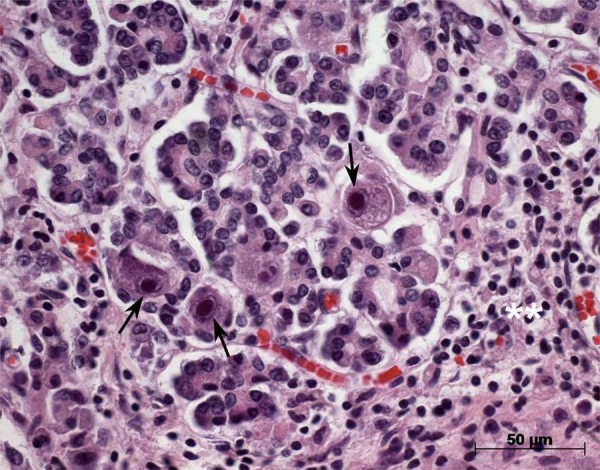
Corte histológico do parênquima pancreático evidenciando discreto infiltrado inflamatório linfocitário (duplo asterisco) e células glandulares com nítida inclusão viral nuclear de padrão citomegálico (setas). Coloração por hematoxilina e eosina.

Ao exame externo do cadáver, notou-se deiscência parcial da sutura da safenectomia, com saída de escassa secreção aos cortes; exame histológico evidenciou extenso processo inflamatório agudo e purulento na derme e hipoderme, com áreas de necrose e presença de microestruturas compatíveis com bactérias degeneradas ( Figura 7 ). O coração pesou 396 g, e notou-se dilatação de ambos os átrios, particularmente do esquerdo. Presença de *patch* de pericárdio medindo 15 mm de diâmetro ocluindo adequadamente comunicação interatrial na fossa oval ( Figura 8 ). A valva mitral exibia plástica da cúspide posterior, com presença de extensa sutura cirúrgica recente com área de reforço; entretanto, era nítida retração de parte da cúspide, com consequente ausência de coaptação adequada ( Figura 8 ). A cúspide anterior exibia discreto espessamento e abaulamento, com cordas tendíneas finas e delicadas. Não havia vegetações. As demais valvas cardíacas não exibiam anormalidades. Havia cirurgia de revascularização recente do miocárdio, com anastomose de artéria mamária na artéria interventricular anterior e ponte de veia safena para o segmento distal da coronária direita, ambas pérvias. Aos cortes transversais dos ventrículos, notou-se discreta miocardioesclerose do esquerdo, inexistindo áreas de infarto agudo. A artéria pulmonar encontrava-se dilatada, com presença de discretas placas ateroscleróticas nos ramos principais. A aorta e artérias coronárias exibiam aterosclerose de grau discreto/moderado, com placas focalmente calcificadas e ulceradas na aorta. O exame dos pulmões evidenciou congestão passiva crônica e extensas áreas de infarto na base do lobo inferior direito, com outras menores em regiões posteriores dos lobos superior e inferior esquerdo. Exame histológico confirmou o diagnóstico de infarto pulmonar, com áreas de aspecto séptico apresentando intenso infiltrado neutrofílico purulento, com presença de microestruturas compatíveis com bactérias degeneradas ( Figura 9 ). O exame do tubo digestivo mostrou áreas granulosas acastanhadas recobrindo de forma multifocal a mucosa do intestino grosso, com exame histológico compatível com colite aguda pseudomembranosa ( Figura 10 ). Outros achados da necropsia foram esteatose hepática difusa, rim vascular com cicatrizes grosseiras e áreas de atrofia parenquimatosa, e discreta pancreatite linfocitária com células parenquimatosas apresentando inclusão viral de padrão citomegálico ( Figura 11 ). O exame do encéfalo não mostrou anormalidades. **(Dr. Luiz Alberto Benvenuti)**

## Diagnósticos anatomopatológicos

Prolapso da valva mitral de etiologia degenerativa, operado, com insuficiência mitral residual; aterosclerose da aorta e artérias coronárias, com cirurgia de revascularização do miocárdio; infecção bacteriana de tecidos moles na região da safenectomia; colite aguda pseudomembranosa; pancreatite por citomegalovírus; esteatose hepática; septicemia com múltiplos infartos pulmonares (causa do óbito). **(Dr. Luiz Alberto Benvenuti)**

## Comentários

O presente caso refere-se a uma mulher de 56 anos de idade que foi submetida a plástica da valva mitral e cirurgia de revascularização do miocárdio cerca de 2 meses e meio antes do óbito. A paciente apresentava insuficiência cardíaca congestiva, prolapso da valva mitral com insuficiência severa^11^ e obstrução coronariana detectada à cineangiocoronariografia, sendo a maior lesão localizada na artéria descendente posterior (obstrução de 90%). Foi submetida a correção cirúrgica da valvopatia (plástica com ressecção quadrangular da cúspide posterior), ocasião em que se verificou a presença de grande comunicação interatrial, medindo 15 mm, na fossa oval. É interessante notar que a associação entre prolapso da valva mitral e comunicação interatrial não é comum, tendo sido relatada muitos anos atrás em trabalho publicado no mesmo periódico.^12^ Após a cirurgia, a paciente evoluiu com insuficiência mitral residual, classificada como de grau moderado aos exames de imagem. Na autopsia verificamos acentuada retração da cúspide posterior na região da plástica, o que impedia a correta coaptação das cúspides e fundamentava a insuficiência residual. Não havia endocardite infecciosa, cuja possibilidade foi cogitada clinicamente e constitui uma das complicações do prolapso valvar.^13^ O fechamento da comunicação interatrial e a revascularização do miocárdio não apresentavam complicações. Deve-se salientar que a coronariopatia era de origem aterosclerótica e não tinha grandes repercussões miocárdicas, havendo apenas discreta miocardiosclerose do ventrículo esquerdo. A paciente apresentou infecção de tecidos moles na região da safenectomia e septicemia com hemocultura positiva para estafilococos, com progressivo agravamento do quadro clínico até o óbito. Na autopsia, confirmamos a presença de infecção aguda purulenta no local da safenectomia, com áreas de necrose. Não foi possível a identificação segura da presença de bactérias, o que certamente se deve à antibioticoterapia prolongada, provável causa da colite aguda pseudomembranosa encontrada. Não houve exame detalhado do sistema venoso do membro inferior, que poderia ter identificado tromboflebite séptica, provável origem dos êmbolos que ocasionaram os infartos pulmonares, considerados a causa terminal do óbito.

Em trabalho de revisão publicado recentemente, trombose venosa profunda e tromboembolia pulmonar foram detectadas em 1,62% e 0,38% de 3 milhões de pacientes submetidos a cirurgia cardíaca e estiveram associadas a maior mortalidade.^14^ Foi achado de autopsia pancreatite por citomegalovírus, que, entretanto, não apresentou repercussões clínicas significativas. É importante salientar que não se tratava de infecção generalizada por citomegalovírus, que foi identificado apenas no parênquima pancreático. A infecção pancreática por citomegalovírus é rara, tendo sido relatados muito poucos casos até o momento, tanto em pacientes imunodeprimidos quanto em imunocompetentes.^14^ **(Dr. Luiz Alberto Benvenuti)**
