# Supplementary material for: Caso 1/2020 - Mulher de 55 Anos com Insuficiência Cardíaca após Internação por Diagnóstico Presumido de Infarto do Miocárdio e Insuficiência da Valva Mitral com Rotura de Corda Tendínea
Source: Arq Bras Cardiol. 2020 May 11;114(4 Suppl 1):47–56. [Article in Portuguese] doi: 10.36660/abc.20200024 (PMC8149113; doi:10.36660/abc.20200024)
Supplement: Translation

# Translation: Case 1/2020 - A 56 Year-Old Woman Developed Heart Failure after a Presumed Diagnosis of Acute Myocardial Infarction and Mitral Valve Regurgitation with Rupture of Chordae Tendineae

**Authors:** Desiderio Favarato, Luiz Alberto Benvenuti

**Affiliations:** Brazil Instituto do Coração (InCor), HC-FMUSP, São Paulo, SP – Brazil

**Keywords:** Heart Failure/physiopathology, Mitral Valve Prolapse/surgery, Myocardial Infarction, Sepsis, Postoperative Care, Shock, Cardiogenic, Renal Insufficiency

A 55-year-old female patient, from the municipality of Carapicuiba, state of São Paulo, had arterial hypertension and started having dyspnea on major exertion a year and a half before hospitalization. In September 2016, she started having severe chest pain that felt like tightness, which was relieved a little with rest, associated with nausea. She sought medical care in her city, was medicated and discharged to go home. In the next morning, she had a recurrence of pain and was hospitalized. During hospitalization, she had a cardiac arrest, which was reversed with electric shocks. The patient remained hospitalized for 10 days and was discharged with a diagnosis of acute myocardial infarction and mitral valve disease.

After hospital discharge, she developed functional class IV (New York Heart Association) dyspnea, sporadic episodes of paroxysmal nocturnal dyspnea and orthopnea, being referred to InCor-HCFMUSP. She was using ASA 100 mg / day; Furosemide 40mg 3xday; Captopril 50mg 3xday; Clopidogrel 75mg once a day; Simvastatin 40mg 1xd.

The physical examination disclosed a heart rate of 102 bpm, blood pressure of 118x86 mmHg, pulmonary auscultation disclosed crackling rales in lung bases, cardiac auscultation showed rhythmic heart sounds with +++ / 6+ mitral systolic murmur; abdominal examination was normal and there was no lower -limb edema.

The echocardiogram (09/28/16) showed aortic diameter measuring 28 mm, left atrium 47 mm, diastolic left ventricle 48 mm and systolic 26 mm, left ventricular ejection fraction of 77%, septal thickness 11 mm and posterior wall thickness, 9 mm. The mitral valve showed partially ruptured chordae tendineae, posterior cusp eversion into the left atrium, with coaptation failure between the cusps and marked eccentric reflux. The tricuspid valve also showed marked reflux. The pulmonary artery systolic pressure was estimated at 62 mm Hg.

Laboratory tests (Nov. 08, 2016) showed 4300000 red blood cells / mm^3^, hemoglobin 12.3 g / dL, hematocrit 38%, 8110 leukocytes / mm^3^, creatinine of 1.75mg / dL, sodium 140 mEq / L, and potassium 3.8 mEq / L.

The electrocardiogram (11/08/16) showed left atrial overload and final intraventricular conduction delay of the stimulus ( Figure 1 ). Marked cardiomegaly and signs of pulmonary congestion were observed on the chest X-ray ( Figure 2 ). Echocardiogram and cardiac catheterization with coronary angiography were requested.

**Figure 1:** ECG (Nov. 08, 2016) showing sinus rhythm, bi-atrial overload and final intraventricular conduction delay.

**Figure 2:** Chest radiography (Nov. 08, 2016) showing pulmonary congestion and marked cardiomegaly.

The coronary angiography (02/08/17) showed a 40% left coronary artery trunk lesion, two lesions in the anterior interventricular branch, 50% in the ostium and 90% in mid-third; a 60% proximal lesion in the circumflex branch and 60% lesion in the mid-third of the right coronary artery. ( Figure 3 )

**Figure 3:** Coronary angiography. Left panel - left coronary artery in RAO view - 80% anterior interventricular injury and 50% proximal circumflex artery; Right panel - right coronary artery - 50% focal lesion.

A surgical procedure to correct valve regurgitation and coronary artery bypass grafting (CABG) surgery were indicated.

Preoperative tests showed: red blood cells 4300000 / mm^3^, hemoglobin 12.4 g / dL, hematocrit 37%, leukocytes 13990 / mm^3^(2% band cells, 80% segmented, 0% eosinophils, 8% lymphocytes and 10% monocytes), platelets 173000 / mm^3^; total cholesterol 158 mg / dL, HDL-C 28 mg / dL, LDL-C 109 mg / dL, triglycerides 103 mg / dL, creatine-phosphokinase (CPK) 2938 U / L, glucose 120 mg / dL, urea 310 mg / dL, creatinine 4.79 mg / dL, sodium 136 mEq, potassium 4.9 mEq / L, Alanine aminotransferase (ALT) 988 U / L, aspartate aminotransferase (AST) 681 U / L; uric acid 27.1 md / dL, glycated hemoglobin 5.4%, Urinalysis with proteinuria of 0.38 g / L and sediment with 14000 epithelial cells / mL, 63000 leukocytes / mL and 4290 hyaline casts / mL. TSH was 6.25 µIU / mL, free T4 was 0.98 mg / dL. Thrombin time (INR) was 1.4; activated partial thromboplastin time (APTT) ratio was 0.96. Serology for hepatitis B and C and for HIV were negative.

Considering these laboratory alterations, the patient was called to the emergency department of InCor (Feb. 23, 2017).

The patient reported that after undergoing cardiac catheterization on February 8, 2017, she received a prescription for atorvastatin and since then she had been progressing with diffuse myalgia and functional class worsening with dyspnea at rest and orthopnea up to 3 days before hospitalization, associated with reduced urinary output and darkened urine. She also reported chest pain in the infra-mammary region, with irradiation to the epigastric region, with worsening at usual efforts, poorly characterized, lasting for hours without improvement factors. She denied fever and cough. She said she had been constipated for 3 days.

On physical examination, the patient was in regular general condition, 2+/4+ skin pallor, hydrated, +/4+ icteric skin, acyanotic and afebrile. Heart rate was 65 bpm, blood pressure was 70x50 mm Hg, oxygen saturation was 90%, with fine crackles in lung bases; auscultation disclosed rhythmic heart sounds, and 3+ / 6+ regurgitation holosystolic murmur in mitral focus; the abdomen was flat and intestinal sounds noises were present, with a palpable liver at 2 cm from the right costal margin; there was no edema or signs of deep vein thrombosis in the lower limbs.

The diagnoses of cardiogenic shock and rhabdomyolysis, acute renal failure, ischemic hepatitis and possible infective endocarditis were made. Norepinephrine, intravenous furosemide 40 mg every 8 hours, and ceftriaxone and oxacillin antibiotics were prescribed.

Laboratory tests (23 Feb 2017) showed hemoglobin 12 g/dL, hematocrit 35%, leukocytes 14230/mm^3^(82% neutrophils, 8% lymphocytes and 10% monocytes), 147000 platelets/m^3^, CK-MB mass 54.5ng/dL, troponin I 0.349 ng/mL; urea 313 mg / dL, creatinine 4.94 mg/dL, AST 938 U / L, ALT 746 U/L, gamma glutamyl transferase (Gamma-GT) 473 U/L, alkaline phosphatase (AP) 279 U/L, total serum proteins 7 g/dL, total bilirubins 1.67 mg/dL, direct bilirubin 1.15 gm/dL, lipase 799 U /L, C-reactive protein (CRP) 98.69 mg/L. Prothrombin time (INR) was 1.4 and the activated partial thromboplastin time (APTT) ratio was 0.96. Urinary analysis showed free hemoglobin ++, leukocytes 32000/mL, erythrocytes 13000/mL, without casts. Gasometry showed a pH of 7.40, pCO_2_of 18.7 mm Hg, pO_2_of 99.9 mmHg, O_2_saturation of 99.9% and bicarbonate level of 11.2 mmol/L. The lactate level was 49 mg / dL.

Blood culture was positive for *Staphylococcus hominis* , sensitive to oxacillin and the urine culture was positive for multisensitive *E.coli* . The antibiotics were then switched to vancomycin, piperacillin and tazobactam on March 3, 2017. In addition to vasoactive drugs, hemodialysis was performed.

The transthoracic echocardiogram did not disclose vegetations, the left ventricular ejection fraction was estimated at 65%, with no change in segmental motility. The mitral valve showed posterior leaflet prolapse, with signs of associated rupture of chordae tendineae. The Doppler study and color flow mapping showed eccentric regurgitation jet of marked degree.

Laboratory tests (March 02, 2017) showed: hemoglobin 9.2 g/dL, hematocrit 28%, leukocytes 12220 / mm^3^(1% band cells, 88% segmented, 5% lymphocytes and 6% monocytes), platelets 123000 / mm^3^, urea 54 mg/dL, creatinine 1.68 mg / dL, sodium 139 mEq / L, potassium 3.0 mEq / L, AST 44 U/L, ALT 160 U/L.

The patient underwent surgery (March 03, 2017) with mitral valve repair and reconstruction and commissurotomy without annuloplasty (quadrangular resection), atrial septal defect closure and left internal mammary artery bypass grafting to the anterior interventricular artery and saphenous vein grafting to the right posterior descending artery, in addition to atrial septal defect closure.

The echocardiogram in the immediate postoperative period (March 03, 2017) disclosed mild mitral regurgitation.

Chest x-ray (March 03, 2017) at the bedside in the immediate postoperative period showed cardiac monitoring electrodes, central venous catheter, pleural drain in the left hemithorax, sternal metal suture, clear lung fields and normal cardiac area.

Biopsy of the mitral valve posterior leaflet showed fibrosis and marked mucoid degeneration of the cusp stroma (B17-0412)

She had a seizure on March 4, 2017 and started treatment with lamotrigine. The cranial tomography showed no alterations.

The echocardiogram (03/20/17) disclosed mitral valve with reduced cusp coaptation, posterior cusp with mild calcification and reduced mobility, and mild regurgitation. There were no images of thrombi in the atria and their appendages or images suggestive of vegetation.

She was discharged on March 29, 2017 and three days later she came to the emergency department complaining of feeding problems, with vomiting episodes, despite the use of ondansetron and diarrhea. She also complained of hoarseness and tinnitus in both ears. She denied dizziness, vertigo, and fever, but reported dyspnea at rest for 1 day.

She was using Amiodarone 200mg 1x /day, AAS 100mg / day, Lamotrigine 25mg / day, Furosemide 40mg 1x / day, Ondansetron 8mg 3x / day, Omeprazole 20mg 1x / day, and Dipyrone 500mg 4x / day.

On physical examination, she showed 3+/ 4+ skin pallor. Blood pressure was 94x68 mmHg and heart rate was 64 bpm; pulmonary auscultation was normal, and the cardiac auscultation disclosed a 2 + / 6 + mitral systolic murmur; abdomen and lower limbs showed no alterations.

Chest x-ray (Apr. 01, 2017) showed para-hilar and right cardiac border condensation foci and blunting basal pleural effusions of the and global +++ cardiomegaly, with unfolding of the left middle arch and dislocated left main bronchus ( Figure 4 ).

**Figure 4:** Chest radiography - right perihilar condensation and right cardiac border, signs of pulmonary congestion and cardiomegaly.

The ECG (Apr. 02, 2017) disclosed sinus rhythm, left atrial overload, right bundle branch block and changes in ventricular repolarization ( Figure 5 ).

**Figure 5:** ECG showing left atrial overload, right bundle branch block.

Laboratory tests disclosed hemoglobin of 12.3 g / dL, hematocrit 37%, leukocytes 4980 / mm^3^, platelets 213000 / mm^3^, C-reactive protein 23.87mg / L, creatinine 1.56 mg / dL, urea 107 mg / dL, sodium 134 meq / L and potassium 3.4 mEq / L.

The echocardiogram (Apr. 04, 2017) disclosed a left ventricle with preserved systolic dimensions and function, without segmental alterations. The mitral valve showed marked regurgitation with reduced posterior cusp mobility, albeit without stenosis. Filamentary structure was observed at the base of the posterior cusp. The tricuspid valve showed marked regurgitation, with alterations of the valve cusps. Pulmonary systolic blood pressure was estimated at 75 mm Hg.

Chest tomography showed a condensation focus in the anterior segment of the right upper lobe and areas of atelectasis and pleural effusion in both lung bases.

The transesophageal echocardiogram (Apr. 07, 2017) was similar to the transthoracic echocardiogram performed on April 04, 2017.

The patient received antibiotic therapy with vancomycin and meropenem and as there was clinical improvement, she was discharged on April 19, 2017.

On May 21, 2017, she returned to the InCor Emergency Department due to worsening dyspnea, now with orthopnea and lower-limb edema. Moreover, she had had anuria for one day. She also complained of daily vomiting and diarrhea since the second hospital discharge. Five days before this hospitalization, she had been treated at an outpatient clinic and, due to suspected pseudomembranous colitis, she received a prescription for ciprofloxacin and metronidazole.

Physical examination disclosed a respiratory rate of 22 breaths per minute, heart rate of 134 bpm, blood pressure was 105x80 mm Hg, oxygen saturation 98% under 2L / min of O_2_. Pulmonary auscultation showed crackling rales up to the middle third; the heart rate rhythm was regular, without murmurs; the abdomen showed a slight distension, with hydro-air noises were present; bilateral +++ / 4+ lower-limb edema, with no signs of deep venous thrombosis.

Chest radiography (May 21, 2017) showed clear lung fields and cardiomegaly ( Figure 6 ).

**Figure 6:** Chest radiography (May 21, 2017) showing clear lungs and cardiomegaly.

Laboratory tests showed hemoglobin 8.2 g/dL, hematocrit 25%; leukocytes 22750 / mm^3^(94% neutrophils, 3% lymphocytes, 3% monocytes), platelets 217000 / mm^3^; urea 147 mg / dL, creatinine 3.21 mg / dL, C-Reactive Protein 49.19 mg / L; sodium 133 mEq / L and potassium 3.7 mEq / L; lactate 34 mg / dL.

She had a seizure episode and received phenytoin. There was asystole which did not respond to resuscitation maneuvers and she died at 10:55 pm on May 21, 2017.

## Clinical aspects

A 55-year-old patient with arterial hypertension and heart failure for one year and with chest pain, had a cardiorespiratory arrest five months before, developed heart failure and mitral regurgitation due to mitral valve prolapse and rupture of chordae tendineae.

The diagnostic impression is that heart failure due to mitral valve disease preceded the episode of precordial pain and severe dyspnea.

As for the episode of precordial pain, it is discussed whether it was really a myocardial infarction or an episode of rupture of chordae tendineae with a sudden worsening of mitral regurgitation and acute pulmonary edema.

The most recent classification of myocardial infarction (the fourth universal definition) introduced a new concept – “myocardial injury” (myocardial injury without infarction) in which there is an elevation of the lesion marker (troponin level), however without constituting an infarction due to the concomitant absence of suggestive clinical picture and electrocardiographic and wall motility alterations. This situation can be found in a large number of clinical conditions – anemia, ventricular tachycardia, heart failure, kidney disease, hypotension and shock, hypoxemia.

The new definition included two types of myocardial injury: the acute one with troponin curve, elevation and fall, and the chronic one, with sustained elevation of troponin.^1^

In the same publication it can be noted that there is a continuum between the “isolated myocardial injury” and type 2 infarction, as the conditions that originate them are the same, depending only on the intensity and occurrence of electrocardiographic and echocardiographic alterations and a clinical picture compatible with the diagnosis of infarction.

In this sense, there is a case report of a patient who sought the Emergency Services for abdominal discomfort and severe dyspnea. The condition was preceded by chest pain 24 hours before the event. The patient already had a previous diagnosis of mitral valve prolapse. There was an increase in troponin levels and nonspecific alterations in ventricular repolarization. The echocardiogram showed severe mitral regurgitation, rupture of chordae tendineae and severe prolapse of half of the posterior cusp. Unlike the current case, there were no coronary angiography alterations. Diagnoses of acute pulmonary edema and severe mitral regurgitation, probably acute, were made due to rupture of the chordae tendineae.^2^

Thus, in the current case, despite the presence of critical lesions in the coronary arteries, the event described as acute myocardial infarction could have been only an increase in cardiac injury markers in the absence of infarction.

Although mitral valve prolapse is generally associated with a low risk of cardiovascular complications, some publications have doubted this assumption. Avierinos et al.,^3^ in a population study in Olmsted County, Minnesota, found moderate or severe mitral regurgitation and left ventricular dysfunction as primary risk factors for cardiovascular mortality, with the first being greater than the latter. Mild mitral regurgitation, left atrial enlargement, a prolapsed cusp, atrial fibrillation and age older than 50 years were considered secondary risk factors. In this study, cardiovascular morbidity was 30%, overall mortality was 19% and cardiovascular mortality 9% in 10 years of follow-up.^3^ In the Framingham study, 25% of patients with mitral valve prolapse developed significant mitral regurgitation or required surgery in a period of 3 to 16 years.^4^

The rupture of the valvar chordae tendineae is the most common cause of acute mitral regurgitation and its most frequent causes are infective endocarditis, myxomatous degeneration and mitral valve prolapse; however, they can occur in rheumatic valve disease, chest trauma and atherosclerotic heart disease.^3 , 5 , 6^

In the current case, tissue changes in the prolapse itself may be the cause of the rupture; however, one should always rule out infectious endocarditis in this type of complication.

The diagnosis of infective endocarditis is based on clinical, laboratory and echocardiographic aspects. Duke’s criteria are the recommended ones. The diagnosis is made in the presence of 2 major criteria or 1 major and 3 minor, or even 5 minor criteria.

The following are considered major criteria: positive blood culture for endocarditis (two cultures within a 12-hour interval or 3 cultures from two samples collected within a 1-hour interval between them for microorganisms commonly related to endocarditis - *Streptococcus viridans* , *S. bovis* , *Staphylococcus aureus* , or HACEK group, or *Coxiella burnetiid* culture. In addition to the blood culture, evidence of endocardial involvement on echocardiogram (preferably transesophageal) is considered as major criteria: oscillating intracardiac mass in the valve or its supporting structures; valve annular abscess, new or intensified regurgitation.

Among the minor criteria are: predisposition – previous valvar heart disease, use of injectable drugs or venous catheters; elevation of inflammation markers; splenomegaly, hematuria; purpura; fever > 38ºC; vascular phenomena (arterial embolism, septic pulmonary infarction, mycotic aneurysm, intracranial or conjunctival hemorrhage and Janeway lesions); immunological phenomena (glomerulonephritis, Osler nodes, Roth’s spots and elevation of the Rheumatoid Factor); positive blood culture of microorganisms not usually associated with endocarditis.^7^

In the current case, no vegetations were detected on the echocardiogram, there was no fever and a staphylococcus strain was identified in the blood culture that is not usually associated with endocarditis. Moreover, there was no evidence of endocarditis in the histopathological analysis of the valve fragments removed during surgery. Thus, the diagnosis of infectious endocarditis can be ruled out.

This patient had rhabdomyolysis with the use of statins or due to severe ischemia, because in addition to the increase in creatine kinase (CK) levels, she also had an increase in liver enzymes suggestive of ischemic hepatitis.

The rhabdomyolysis presentation was the classic one, with the presence of muscle pain, weakness, darkened urine and marked increase in creatine kinase (CK) levels. Also, its most common complication, acute renal failure, was present.

Simvastatin and atorvastatin are metabolized by CYP3A4 (the most common cytochrome P450 isoenzyme), while rosuvastatin is metabolized by CYP2A9. Thus, the former are more susceptible to drug interactions that increase plasma concentrations and the probability of toxicity. Muscle symptoms are complaints that range from 1% to 10% of patients using statins; however, there is an increase in CK levels in less than 1%.^8^

Ischemic hepatitis is characterized by cardiopulmonary or circulatory failure associated or not with arterial hypotension, massive and reversible elevation of liver aminotransferase enzymes (AST and ALT) and exclusion of other causes of severe liver damage, such as acetaminophen poisoning, viral hepatitis or another type of toxic hepatitis. In the current case, liver damage caused by statins cannot be ruled out and there was no increase in prothrombin times with iNR > 1.5 and the APTT time ratio was normal, alterations present in ischemic hepatitis.^9^

The final stage of the disease of this patient was due to septicemia, which could be due to infection by toxin-producing *Clostridium difficile* . Only toxin-B producing *C. difficile* (TBcd) strains cause infection; however, some strains also produce toxin A (TAcd). They act by inactivating the Rho GTPases pathway through the glycosylation of the threonine residue, which leads to actin depolymerization and cell death and stimulates the inflammation cascade responsible for major tissue damage, diarrhea and pseudomembranous colitis.

The use of antibiotics can lead to an imbalance of the intestinal microbiome with a decrease in Bacteroides and Firmicutes, allowing the proliferation of *Clostridium difficile* . Remember that the patient received broad spectrum antibiotics for a prolonged period.^10^ **(Dr.** **Desiderio Favarato)** .

**Diagnostic hypothesis:** mitral regurgitation due to ruptured chordae tendineae in mitral valve prolapse, septicemia and multiple organ failure. **(Dr.** **Desiderio Favarato)** .

## Necropsy

On external examination of the corpse, partial dehiscence of the saphenectomy suture was noted, with little secretion from the surgical wound; the histological examination disclosed an extensive acute purulent inflammatory process in the dermis and hypodermis, with areas of necrosis and the presence of microstructures compatible with degenerated bacteria ( Figure 7 ). The heart weighed 396 g, with dilation in both atria, particularly the left one. Presence of a pericardium patch measuring 15 mm in diameter, adequately occluding the atrial septal defect in the oval fossa ( Figure 8 ). The mitral valve showed posterior cusp repair, with the presence of recent extensive surgical sutures with a reinforcement area; however, there was a clear retraction of part of the cusp, with a consequent absence of adequate coaptation ( Figure 8 ). The anterior cusp showed slight thickening and bulging, with thin and delicate chordae tendineae. There were no vegetations. The other cardiac valves showed no abnormalities. There was evidence of recent CABG surgery, with anastomosis of the mammary artery to the anterior interventricular artery and a saphenous vein graft to the distal segment of the right coronary artery, both patent. Cross- sections of the ventricles showed mild left myocardiosclerosis, with no areas of acute infarction. The pulmonary artery was dilated, with the presence of discrete atherosclerotic plaques in the main branches. The aorta and coronary arteries showed mild / moderate atherosclerosis, with focally calcified and ulcerated plaques in the first. Lung examination showed chronic passive congestion and extensive infarction areas at the base of the right lower lobe, with smaller ones in posterior regions of the upper and lower left lobes. The histological examination confirmed the diagnosis of pulmonary infarction, with areas of septic aspect showing intense purulent neutrophilic infiltrate, with the presence of microstructures compatible with degenerated bacteria ( Figure 9 ).

**Figure 7:** Histological section of the subcutaneous tissue in the saphenectomy suture region showing an intense purulent inflammatory process with areas of tissue necrosis. Hematoxylin-eosin staining.

**Figure 8:** View of the left open atrium and mitral valve. Note the patch adequately closing the large atrial septal defect (asterisk), and surgical suture on the posterior cusp with an extensive retraction area (arrow) that prevents adequate cusp coaptation.

**Figure 9:** Histological sections of the lungs showing an area of pulmonary infarction (A), with obliteration of the tissue structure, leakage of fibrin and hemorrhage, and an area of septic infarction (B), with extensive purulent inflammatory infiltrate similar to that shown in Figure 7. Hematoxylin-eosin staining.

Examination of the digestive tract showed multifocal brownish granular areas covering the mucosa of the large intestine, with histological examination compatible with acute pseudomembranous colitis ( Figure 10 ). Other necropsy findings were diffuse liver steatosis, vascular kidney with renal scarring and areas of parenchymal atrophy, and mild lymphocytic pancreatitis with parenchymal cells showing viral inclusion with a cytomegalic pattern ( Figure 11 ). The examination of the brain showed no abnormalities. **(Dr. Luiz Alberto Benvenuti)**

**Figura 10:** Macroscopic view of the colon with multiple brownish and granular pseudomembranes (arrows) covering the mucosa in a multifocal manner.

**Figura 11:** Histological section of the pancreatic parenchyma showing a mild lymphocytic inflammatory infiltrate (double asterisk) and glandular cells with clear nuclear viral inclusion with a cytomegalic pattern (arrows). Hematoxylin-eosin staining.

## Anatomopathological diagnoses

Operated degenerative mitral valve prolapse, with residual mitral regurgitation; atherosclerosis of the aorta and coronary arteries, with CABG surgery; soft tissue bacterial infection in the saphenectomy region; acute pseudomembranous colitis; pancreatitis due to cytomegalovirus; hepatic steatosis; septicemia with multiple pulmonary infarctions (cause of death). **(Dr. Luiz Alberto Benvenuti)**

## Comments

The present case refers to a 56-year-old woman submitted to mitral valve surgery and CABG surgery approximately 2 ½ months before death. The patient had congestive heart failure, mitral valve prolapse with severe regurgitation^11^ and coronary obstruction detected by coronary angiography, with the largest lesion located in the posterior descending artery (90% obstruction). She underwent surgical correction of valvar heart disease (repair with quadrangular resection of the posterior cusp), on which occasion the presence of a large atrial septal defect was observed in the oval fossa, measuring 15 mm. It is noteworthy that the association between mitral valve prolapse and atrial septal defect is not common, having been reported many years ago in a study published in this same journal.^12^ After surgery, the patient developed residual mitral regurgitation, classified as moderate on imaging exams. At the autopsy, a marked retraction of the posterior cusp in the repair region was observed, which prevented the adequate coaptation of the cusps and justified the residual regurgitation. There was no infectious endocarditis, which was clinically considered and consists of one of the complications of valve prolapse.^13^ The closure of the atrial septal defect and CABG did not have any complications. It should be noted that coronary artery disease was of atherosclerotic origin and did not have any major myocardial consequences, with only mild left ventricular myocardiosclerosis. The patient had soft tissue infection in the saphenectomy region and septicemia with positive blood culture for staphylococci, with progressive worsening of the clinical picture until death. At autopsy, we confirmed the acute purulent infection at the saphenectomy site, with areas of necrosis.It was not possible to safely identify the presence of bacteria, which is certainly due to prolonged antibiotic therapy, a probable cause of the acute pseudomembranous colitis. There was no detailed examination of the lower-limb venous system, which could have identified septic thrombophlebitis, probable origin of the emboli that caused pulmonary infarctions, considered the final cause of death.

In a recently published review, deep vein thrombosis and pulmonary thromboembolism were detected in 1.62% and 0.38% of 3 million patients undergoing cardiac surgery and were associated with higher mortality.^14^ Pancreatitis caused by cytomegalovirus was found at the autopsy, which, however, did not have significant clinical consequences. It is important to note that this was not a generalized cytomegalovirus infection, which was identified only in the pancreatic parenchyma. Pancreatic infection by cytomegalovirus is rare, and very few cases have been reported so far, both in immunocompromised and immunocompetent patients.^14^ **(Dr. Luiz Alberto Benvenuti)**
